# *BRAF* V600E mutations in urine and plasma cell-free DNA from patients with Erdheim-Chester disease

**DOI:** 10.18632/oncotarget.1964

**Published:** 2014-05-13

**Authors:** Filip Janku, Cecile Rose T. Vibat, Karena Kosco, Veronica R. Holley, Goran Cabrilo, Funda Meric-Bernstam, Vanda M. Stepanek, Patrick P. Lin, Lorieta Leppin, Latifa Hassaine, Jason C. Poole, Razelle Kurzrock, Mark G. Erlander

**Affiliations:** ^1^ Department of Investigational Cancer Therapeutics, The University of Texas MD Anderson Cancer Center; ^2^ Trovagene Inc. San Diego, CA; ^3^ Department of Orthopedic Oncology, The University of Texas MD Anderson Cancer Center; ^4^ Moores Cancer Center, The University of California San Diego, La Jolla, CA

**Keywords:** BRAF, cell-free DNA, plasma, urine, Erdheim-Chester disease

## Abstract

Erdheim-Chester disease (ECD) is a rare histiocytosis with a high prevalence of *BRAF* V600E mutation (>50% of patients). Patients with *BRAF*-mutant ECD can respond to BRAF inhibitors. Unfortunately, the lack of adequate archival tissue often precludes *BRAF* testing. We hypothesized that cell-free DNA (cfDNA) from plasma or urine can offer an alternative source of biologic material for testing. We tested for *BRAF* V600E mutation in cfDNA from the plasma and urine of 6 ECD patients. In patients with available archival tissue, the result of *BRAF* mutation analysis was concordant with plasma and urine cfDNA results in all 3 patients (100% agreement, kappa 1.00). In all 6 patients, *BRAF* mutation analysis of plasma and urine cfDNA was concordant in 5 of 6 patients (83% agreement, kappa 0.67). Testing for *BRAF* V600E mutation in plasma and urine cfDNA should be further investigated as an alternative to archival tissue mutation analysis.

## INTRODUCTION

Erdheim-Chester disease (ECD) is a rare form of non-Langerhans cell histiocytosis affecting adults, which is associated with xanthogranulomatous infiltration of foamy macrophages.[1-3] ECD is deemed to be driven by increased signaling within the mitogen-activated-protein kinase pathway. Advances in genome sequencing technologies led to the identification of *BRAF* V600E mutations in at least 50% of patients with ECD.[4] Furthermore, preliminary results suggest that patients with ECD and *BRAF* mutations can benefit from targeted inhibition of BRAF protein with BRAF inhibitors.[5] Unfortunately, archival tissue often does not provide an adequate amount of DNA for molecular testing. Therefore, novel technologies allowing mutation analysis to be performed using alternative sources of biologic material are needed.[6]

Cell-free DNA (cfDNA) is released to the circulation from cells undergoing apoptosis, necroptosis and active secretion and has been identified in the plasma and urine of patients with cancer.[7, 8] Detecting and quantifying the amount of mutant cfDNA fragments harboring specific mutations can be used as an alternative to tissue testing. Some data suggest that the amount of mutant DNA correlates with tumor burden and can be used to identify the emergence of resistant mutations.[9-14] The concept of mutation testing from urine cfDNA has been assessed in a pilot study in patients with advanced colorectal cancer and other colorectal diseases in which *KRAS* mutations in urine cfDNA were concordant in 95% of cases with *KRAS* mutation status in tumor tissue.[15] We examine in our study whether urine and plasma cfDNA can be used as an alternative to tissue biopsies for *BRAF V600E* mutation testing in patients with ECD.

## RESULTS AND DISCUSSION

A total of 6 patients with ECD were enrolled. Their median age at diagnosis was 46 years (range, 26 to 71 years) and most patients were white 4 (67%) and male 4 (67%). Tumor tissue *BRAF* V600E mutation testing with targeted next-generation sequencing and/or allele-specific PCR in the CLIA-certified laboratory was requested for all 6 patients, but 3 patients had insufficient tissue samples, precluding mutation analysis (Table 1). *BRAF* V600E mutations were detected in 2 (67%) of 3 tested tumor tissue samples, 3 (50%) of 6 plasma cfDNA samples and 4 (67%) of urine cfDNA samples (Figure 1). Observed agreements were 100% (3 of 3, kappa 1.00) for tumor tissue and plasma cfDNA, 100% (3 of 3, kappa 1.00) for tumor tissue and urine cfDNA, and 83% (5 of 6, kappa 0.67) for plasma cfDNA and urine cfDNA (Table 1). In addition, there were no *BRAF* V600E mutations in plasma and urine cfDNA from 14 patients with metastatic cancer with confirmed wt *BRAF* in their tumor tissue (data not shown). Finally, only one patient (patient 1) was treated with a *BRAF* inhibitor; however, the treatment outcomes were not available at the time of analysis.

**Table 1:** Urine and plasma cell-free DNA *BRAF* VGOOE mutations

Patient#	Age at diagnosis	Gender	Involvement	Urine *BRAF* VGOOE/WT	Plasma *BRAF* VGOOE/WT	Patient Tissue *BRAFstatus*
1	59	Male	CNS, cardiac, bones, renal	V600E (22.590%)	V600E (8.598%)	V600E
2*	43	Male	CNS, bones, renal	V600E (0.311%)	V600E (1.522%)	V600E
3	26	Male	Skin	Wild-type (0.010%)	Wild-type (0.063%)	Wild-type
4	71	Female	Bones, lymph nodes	V600E (0.159%)	Wild-type (0.047%)	Unknown**
5	43	Female	CNS, bones	V600E (4.940%)	V600E (0.261%)	Unknown**
6	49	Male	CNS, cardiac, omentum, retroperitoneum	Wild-type indeterminate (0.079%)	Wild-type (0.048%)	Unknown**

CNS; central nervous system

* Urine and plasma collected on different dates

** lnsuficient tissue for molecular analysis

**Figure:**
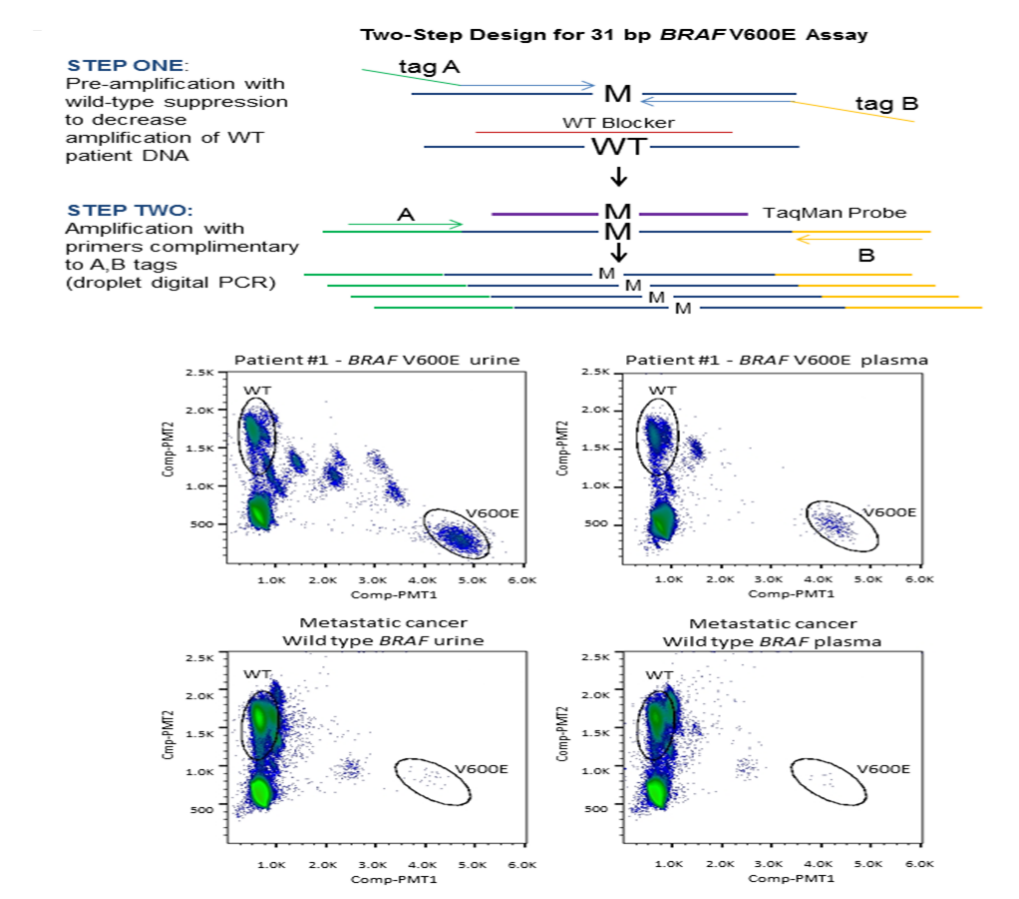


*BRAF* V600E mutations have been reported in more than 50% of patients with ECD.[4] In addition, preliminary data reveal encouraging activity of *BRAF* inhibitors such as vemurafenib in ECD patients with *BRAF* V600E mutations.[5] Mutation analysis of tumor tissue remains a gold standard for molecular analysis; however, in some disorders such as ECD, available tumor tissue often does not provide enough DNA for molecular analysis. In our experience, archival tissue testing for *BRAF* mutations is not feasible in up to 60% of patients.[6] This creates a major hurdle for further implementation of personalized therapies into the ECD therapeutic armamentarium since *BRAF* inhibitors in general can be effective in patients with *BRAF* mutations but detrimental in patients without them.[16] Therefore, there is a clear need for a new and easily obtainable source of material that can be used to analyze tumor molecular aberrations. [7, 8] cfDNA is released to the circulation from cells undergoing apoptosis, necroptosis and active secretion and has been identified in the plasma or urine of patients with cancer.[9-14] Arguably, cfDNA can originate from multiple tumor sites and its molecular analysis may perhaps better reflect prevailing molecular aberrations.[9, 10]

Our study suggests that mutation analysis of plasma and/or urine cfDNA from patients with ECD can be concordant with archival tissue and should be investigated as its alternative in furthering personalized therapy for patients whose tumor tissue is in short supply.

## METHODS

Patients with ECD were referred to the Clinical Center for Targeted Therapy at The University of Texas MD Anderson Cancer Center (MD Anderson) and prospectively enrolled starting in January 2013. In addition, 14 patients with metastatic cancers with known wild-type (wt) *BRAF* in tumor tissue were used as a negative control group. Database registration of patients and pathology assessment were performed at MD Anderson. The study and all treatments were conducted in accordance with MD Anderson Institutional Review Board guidelines. A total of 10mL blood samples and approximately 60-120mL of urine from each consented patient were used for DNA isolation.

Urine cfDNA was isolated by adding urine to an ion-exchange resin (GE Healthcare; Pittsburgh, PA). Nucleic acid was eluted with a chaotropic agent and subsequently purified by a silica-based resin (QIAGEN; Germantown, MD). The eluate was further purified using a specific molecular cutoff filter concentrator (Millipore; Billerica, MA), followed by a size exclusion column (Bio-Rad; Hercules, CA). Plasma cfDNA was isolated using the QIAamp Circulating Nucleic Acid Kit (QIAGEN; Germantown, MD) according to the manufacturer's instructions.

Urine and plasma cfDNA were quantified by a droplet digital PCR (ddPCR; QX-100, BioRad; Hercules, CA) assay to a 44bp amplicon of *RNase* P, a single-copy gene. Quantified DNA (12.4ng to 60ng) was used for a two-step PCR assay for rare mutant allele detection of a 31bp region containing *BRAF* V600E (Figure 1). The first step involved pre-amplification with two primers flanking the *BRAF* V600E locus, where both primers contain non-complementary 5' tags which hybridize to second round primers. A complementary blocking oligonucleotide suppressed wt *BRAF* amplification, achieving enrichment of the mutant *BRAF* V600E sequence within the pre-amplification step. The second step entailed a duplex ddPCR reaction using FAM (V600E *BRAF*) and VIC (wt *BRAF*) TaqMan probes to enable differentiation of mutant versus wild-type quantification, respectively. The RainDrop ddPCR instrument (RainDance; Billerica, MA) was used for PCR droplet separation, fluorescent reading, and counting droplets containing mutant sequence, wt sequence, or unreacted probe. For a given patient sample, the assay reported *BRAF* V600E mutation fragments detected as a percentage of detected wt *BRAF*. Previous to this study, accuracy of the urine-based ddPCR *BRAF* V600E assay was verified in 89 urine specimens from 50 healthy control samples (Precision Med; Solana Beach, CA) and 39 samples from 20 patients with known positive *BRAF* V600E mutation tissue biopsies as determined in a CLIA laboratory.[17] Thresholds for mutation detection in urine were determined by assessing these data using a classification tree. Minimizing the percentage of false negatives was given a higher importance than minimizing false positives. Thresholds were defined as no detection – wt (<0.05%), indeterminate (0.05% - 0.107%), and detected – V600E (>0.107%). For plasma detection, plasma from 13 patients with wt *BRAF* metastatic cancer was used to determine a threshold for detection of *BRAF* V600E mutations. For plasma, >0.094% mutant, equivalent to three standard deviations (0.021%) above the mean of wt *BRAF* controls (0.031%), was considered positive for *BRAF* V600E mutation.
